# A Study on the Correlation Between Quality of Life and Unhealthy Emotion Among Patients With Endometriosis

**DOI:** 10.3389/fpsyg.2022.830698

**Published:** 2022-03-14

**Authors:** Guimei He, Jiebing Chen, Zhangqing Peng, Kai Feng, Chunqi Luo, Xun Zeng

**Affiliations:** The First Affiliated Hospital, Sun Yat-sen University, Guangzhou, China

**Keywords:** endometriosis, anxiety, depression, quality of life (QOL), influencing factors

## Abstract

**Objective:**

To investigate the influence of quality of life (QOL) on unhealthy emotions as well as relevant factors among patients with endometriosis for supporting relevant clinical care.

**Methods:**

A convenience sampling method was used to administer questionnaires to 139 patients with endometriosis, using the Hamilton Anxiety Inventory (HAMA), the Depression Anxiety Scale (SDS), and the SF-12 Health Survey Short Form, and the results were analyzed. The SPSS20.0 software was used for statistical analysis on relevant data. If *P* < 0.05, there was statistical significance.

**Results:**

Twelve-Item Short Form (SF-12) for health survey covered two comprehensive indexes, i.e., physical component summary (PCS) and mental component summary (MCS) scores. MCS score was the main factor influencing anxiety and depression in patients with endometriosis; the higher the MCS score, the lower the anxiety and depression degrees in patients with endometriosis (*OR* = 0.912, 95% CI: 0.877–0.949; *OR* = 0.899, 95% CI: 0.866–0.933). PCS score was a factor influencing anxiety degree; the higher the PCS score, the lower the anxiety degree (*OR* = 0.936, 95% CI: 0.891–0.983).

**Conclusion:**

The QOL of patients with endometriosis is negatively correlated with anxiety and depression. Therefore, improvement in QOL may help relevant patients to relieve their unhealthy emotions.

## Introduction

The aim of our study is to investigate the influence of quality of life (QOL) on unhealthy emotions as well as relevant factors among patients with endometriosis. Endometriosis is defined as growth of endometrial gland and/or stroma growing in a site outside the uterine cavity (Zondervan et al., 2018). It is estimated that endometriosis affects approximately 10% of women of childbearing age, and the need for surgery to definitively diagnose endometriosis, along with other factors such as demographic sample, lead to some variations in prevalence estimates. A recent Australian study showed that 1 in 9 women were clinically diagnosed with endometriosis after following 13,508 patients for 20 years, with the majority being diagnosed in their 30s (Rowlands et al., 2021). Lesions either inside or outside the pelvic/abdominal cavity, involving different tissues and organs, can cause a series of symptoms and signs, for example, dysmenorrhea, menstrual disorders, infertility, and dyspareunia (Vercellini et al., 2014). Symptoms of endometriosis often affect psychological and social functioning of patients (Vitale et al., 2016, 2017; Lagana et al., 2017). For example, dysmenorrhea is the main clinical manifestation of endometriosis, and studies have shown that dysmenorrhea has significant effects on physical QOL and mental health. Psychological intervention is recommended while medical treatment of endometriosis with pain may not be sufficient, but there are limited data on therapeutic effects of psychological interventions (Facchin et al., 2015). Also, endometriosis may affect marital relationships, work, and social life, for it causes infertility, periodic pain, etc. Patients may have mental and emotional disorders due to anxiety, fear, and low self-esteem, which compromise their QOL. QOL refers to a person’s sense of his/her place in the corresponding cultural and value system including and exceeding the concept of health (Ferrans, 1990), covering the living environment, psychology, and social support. It can reflect the health status of individuals or groups from multiple dimensions.

## Materials and Methods

### Subjects

This is a research study that includes in-patients diagnosed with endometriosis at the First Affiliated Hospital of Sun Yat-sen University between July 2020 and April 2021. Inclusion criteria for the study were: no previous mental illness or history of psychiatric diagnosis, no neurological disease, no other chronic diseases, patients with stable conditions who can complete the questionnaire independently and voluntarily, and patients must be histologically diagnosed as having endometriosis. These patients underwent surgery after being clinically diagnosed with endometriosis based on symptoms and ultrasound results. Exclusion criteria of the study were: patients with serious audiovisual impairment that may influence the understanding and response to the questionnaire, patients who suffer from serious mental illness, and patients who cannot cooperate or refuse to cooperate.

### Methods

The self-designed scale for general information survey, 12-item Short-Form (SF-12), Hamilton Anxiety Scale (HAMA), and Self-rating Depression Scale (SDS) were used to investigate patients with endometriosis. General information on the patients included age, education level, family concern level, obstetrical history, dysmenorrhea, dyspareunia, use of painkiller, and efficacy of painkiller. HAMA was assessed by surveyors who were professionally trained and qualified. Other questionnaires were completed by the subjects independently after explanation by the investigators. The investigators then checked all the questionnaires, so as to ensure the authenticity and validity of the survey results.

#### Method for Quality of Life Evaluation

Twelve-Item Short Form (SF-12) is a short form for health survey that includes 12 items covering 8 dimensions: general health (GH), physiological function (PF), role-physical (RP), bodily pain (BP), mental health (MH), vitality (VT), social function (SF), and role-emotional (RE). The SF-12 was summarized as two comprehensive indexes, namely, physical component summary (PCS) and mental component summary (MCS) scores. A scoring algorithm ranging from 0 to 100 was used to calculate body functional component score and mental functional component score. The higher the scores, the better the self-perceived QOL; the lower the scores, the poorer the outcomes (Busija et al., 2011). The physical component summary (PCS) and mental component summary (MCS) scores worked out by SF-12, a simplified version of SF-36, are equivalent to the PCS and MCS scores worked out by SF-36. SF12 is more concise and efficient than SF-36, so it is widely used in surveys of large-scale populations (Kim et al., 2017).

#### Method for Emotional State Evaluation

Hamilton Anxiety Scale (HAMA) (Lu et al., 2020) covered 14 items, such as anxiety, tension, fear, and sensory system, and for each item, the score was between 0 and 4, which represented five levels: 0 represented no symptoms, 1 represented mild, 2 represented moderate, 3 represented severe, and 4 represented extremely severe. A total score of 29 or above indicated serious anxiety, 21 or above obvious anxiety, 14 or above definite anxiety, 7 or above possible anxiety, and below 7 normal condition. Self-Rating Depression Scale (SDS) (Zung, 1965, 1971) used a 4-point Likert scale covering 20 items, with 1, 2, 3, and 4 indicating “occasional or none,” “sometimes,” “often,” and “persistent,” respectively. Scores for all of the items were added and multiplied by 1.25, thus obtaining a total score. For the total score, the critical value was 53, and a total score above 53 indicated depression. The higher the total score, the more serious the symptom: 53–62 indicated mild depression, 63–72 indicated moderate depression, and 73 or above indicated severe depression.

### Statistical Analysis

For quantitative indexes, analysis of variance was performed, while for qualitative indexes, Kruskal-Wallis H test was adopted. Ordered logistic regression was utilized for a multifactor model. If *P* < 0.05, there was statistical significance. The SPSS 20.0 software was used for statistical analysis.

### Ethics Statement

This study has been approved by the Ethics Committee of the First Affiliated Hospital of Sun Yat-sen University, with approval number: Lun Shen [2021] No. 237. Each patient signed an informed consent form.

## Results

### General Features of Included Patients

Univariate analysis on anxiety and depression in patients with endometriosis: the results showed that PCS and MCS scores were factors influencing anxiety level. The higher the PCS score, the lower the anxiety level (*OR* = 0.942, 95% CI: 0.97–0.977), and the higher the MCS score, the lower the anxiety level (*OR* = 0.905, 95% CI: 0.877–0.934). The MCS score was the only factor influencing depression level. The higher the MCS score, the lower the depression level (*OR* = 0.899, 95% CI: 0.866–0.933) (see Tables 2, 3).

**TABLE 1:** General characteristics of in-patients with endometriosis.

Variables		N (%)
Age	<30	28 (20.1)
	30–39	62 (44.6)
	≥40	49 (35.3)
	x ±s	35.8 ± 6.79
Education level	Primary school or below	2 (1.4)
	Secondary school or technical school	39 (28.1)
	Bachelor degree or above	88 (63.3)
	Master degree or above	10 (7.2)
Family concern level	Care	120 (86.3)
	General/living alone/indifferent	19 (13.7)
Obstetrical history	Married, with at least a child	70 (50.4)
	Married, without child	31 (22.3)
	Unmarried, without child	33 (23.7)
	Divorced	5 (3.6)
Dysmenorrhea	Yes	118 (84.9)
	No/no sex	21 (15.1)
Taking painkiller for dysmenorrhea	Yes	68 (48.9)
	No	71 (51.1)
Dyspareunia	Yes	56 (40.3)
	No/	83 (59.7)
PCS	x ±s	42.0 ± 8.99
MCS	x ±s	44.0 ± 11.91
Anxiety level	Without anxiety	42 (30.2)
	Possible anxiety	43 (30.9)
	Definite anxiety	29 (20.9)
	Obvious anxiety	12 (8.6)
	Potential serious anxiety	13 (9.4)
Depression level	Without depression	88 (63.3)
	Mild depression	30 (21.6)
	Moderate to severe depression	21 (15.1)

*PCS, physical component summary; MCS, mental component summary.*

**TABLE 2:** Univariate analysis and multivariate analysis on anxiety in patients with endometriosis.

Variable	Grouping	Anxiety level (n)		Univariate analysis	Multivariate analysis	
		Without anxiety/possible anxiety	Definite anxiety/obvious anxiety/potential serious anxiety	*P*-value	OR	95%CI
Family concern level	General/living alone/indifferent	6	13	0.004	0.442	0.12–1.628
	Care	79	41			
Dyspareunia	Yes	27	29	0.088	1.984	0.846–4.655
	No/no sex	58	25			
PCS	$\bar{\text{x}}$ ± s	44.3 ± 8.66	38.4 ± 8.37	<0.001	0.936	0.891–0.983
MCS	$\bar{\text{x}}$ ± s	48.8 ± 9.43	36.4 ± 11.55	<0.001	0.912	0.877–0.949

*PCS, physical component summary; MCS, mental component summary; OR, odds ratio; CI, confidence interval.*

**TABLE 3:** Univariate analysis and multivariate analysis on depression in patients with endometriosis.

Variable	Grouping	Degree of depression (n)			Univariate analysis	Multivariate analysis	
		Without depression	Mild depression	Moderate to severe depression	*P*-value	OR	95%CI
Family concern level	Care	83	20	17			
	General/living alone/indifferent	5	10	4	0.001	0.599	0.214–1.679
PCS	$\bar{\text{X}}$ + s	43.5 ± 8.95	38.9 ± 7.99	39.9 ± 9.38	0.026	0.975	0.933–1.020
MCS	$\bar{\text{X}}$ + s	48.8 ± 8.58	38.4 ± 11.96	31.5 ± 11.7	<0.001	0.899	0.866–0.933

*PCS, physical component summary; MCS, mental component summary; OR, odds ratio; CI, confidence interval.*

For patients with endometriosis, the higher the QOL, the lower the anxiety and depression levels. Physical component summary (PCS) and mental component summary (MCS) scores are two comprehensive indexes of SF-12: the higher the PCS and MCS scores, the lower the anxiety and depression levels. At present, there is no report concerning the correlation between QOL and unhealthy emotion worldwide. For patients with endometriosis, the PCS score (x ±s) was 42 ± 8.99, and the MCS score (x ±s) was 44. ± 11.91. The PCS score was obtained by a calculation based on general health (GH), physiological function (PF), role-physical (RP), and bodily pain (BP) scores, and MCS score was obtained by a calculation based on mental health (MH), vitality (VT), social function (SF), and role-emotional (RE) scores (Lin et al., 2020).

## Discussion

Worldwide, most studies on the QOL of patients with endometriosis have focused only on its relevant factors and neglected the role of emotions. This study is the first attempt to investigate the correlation between unhealthy emotion and QOL among patients with endometriosis, aiming to improve psychological care in nursing, as well as the QOL of patients with endometriosis in the future.

In our study, about 84.9% of the patients with endometriosis had dysmenorrhea (see Table 1), which was different from the corresponding result of a multicenter cross-sectional study (with 1,418 patients enrolled), i.e., 60.4%, but the difference was possibly related to the inclusion and exclusion criteria and difference in sample size. Overall, our study and that multicenter cross-sectional study both have shown that the proportion of patients with dysmenorrhea for endometriosis is relatively high; therefore, it is noteworthy (Nnoaham et al., 2011). In this study, only 30.2% of the patients with endometriosis had no anxiety, and 18% of the patients had obvious anxiety or potential serious anxiety. Depression was observed in 36.7% of the patients, including 15.1% with moderate to severe depression or more serious depression, which was consistent with the study of Warzecha et al. (2020). A prospective cohort study of Laganà et al. (2015) found that depression, anxiety, and sensitivity in patients with endometriosis were significantly higher than in the control group. Interestingly, the investigators adopted patients who had undergone surgery for benign adnexa as the control group. Therefore, mental problems like anxiety and depression in patients with endometriosis will be far more serious than in normal people (Laganà et al., 2015). In this case, the influence of endometriosis on relevant mental conditions may be more serious than expected (Qin et al., 2018). Pain was learned by study team members through interviews with enrolled patients. Dysmenorrhea may bring endless harm to the patients’ body, and make patients fear the onset of their menstruation. The painful experience of menstruation instills fear in them, and 48.9% of them need to take painkillers for it. Drugs can help 26.6% of patients to relieve the pain, but the analgesic effect is poor in 22.3% of the patients, whose QOL, social activity, and work may be compromised. Studies have shown that among women with endometriosis, especially those with dysmenorrhea, the risk of mental disorders such as anxiety and depression is increased (Chen et al., 2016). Long-term physical pain may affect patients’ PF and RP, and clinically, patients’ PCS score may be increased by improving their GH, PF, and RP, and alleviating their BP, while their MCS score may be increased by improvement in MH, VT, SF, and RE. In a word, for them, both physical and mental conditions should be paid attention to, so as to improve their QOL.

The univariate analysis indicated that for the influence of family concern level on anxiety and depression of patients, there was statistical significance (*P* < 0.05). Relative to incidence and mortality of cervical cancer and ovarian cancer as gynecological diseases, endometriosis failed to attract public attention, and there are only few studies on family support and social support for patients with endometriosis. For patients with endometriosis, dysmenorrhea is often the first symptom for treatment, and, usually, as they come to hospital for this, the pain can no longer be relieved by oral painkillers. Many families even think that women’s dysmenorrhea is normal, and a painkiller is enough for it. Therefore, they cannot comfort patients as they need, which makes patients feel helpless. At the same time, for patients with endometriosis, relevant diagnosis and treatment are time-consuming. Duration may be a few years, or even 30 years, which may make patients’ families become accustomed to the condition. Thus, the loneliness of patients may become more serious because of lack of family care. As a common cause of infertility, endometriosis may lead to infertility in approximately 20–40% of relevant patients (Ozkan et al., 2008). Infertility makes patients feel guilty to their families and their husbands, which may increase patients’ psychological burden. Therefore, during care for patients with endometriosis, their family relationships and social support systems should also be further understood. In providing patients with knowledge of relevant diseases, their families should also be invited to learn about endometriosis, postoperative drug management, prevention of recurrence, etc. At the same time, the significance of family care and support to the mental health of patients should be emphasized. Patients and their families should be encouraged, or patients’ families should be encouraged to take the initiative to communicate with the patients, so as to strengthen their families’ support and care, make the patients have a sense of belonging and cope with the disease actively. Also, relevant clinical data should be collected, and cases of successful birth after surgery should be introduced as references for enhancing the confidence of patients and their families in relevant treatment.

The univariate analysis found that for the influence of dyspareunia on anxiety of patients, there was statistical significance (*P* < 0.05). In this study, 40.3% of the patients were accompanied with dyspareunia, and a prospective study of Shum et al. (2018) found that half of patients with endometriosis had dyspareunia. In patients with endometriosis, painful nodules may be palpable at the sacral ligament, Douglas cul-de-sac, and vaginal vault (Yang et al., 2021). In sexual life, dyspareunia is due to increased tension and displacement of the sacral ligaments, which are less elastic. In addition, Zoladex and dienogest may decrease patients’ hormone levels during relevant treatment, resulting in vaginal dryness, which may cause dyspareunia during intercourse. Dyspareunia may decrease the satisfaction relevant to couples’ sexual lives, and increase patients’ sexual psychological disorder. As the duration of the disease is prolonged, the harmony between husbands and wives, family stability, QOL, and quality of sexual life may be compromised, thus aggravating patients’ unhealthy emotions. Increased psychological burden in sexual life may further aggravate sexual life, thus forming a vicious cycle. There are several ways to treat endometriosis-related pelvic pain, with medication being the main treatment (Vercellini et al., 2018). Caruso et al. (2019) pointed out that endometriosis-related pain has a negative impact on QOL as well as sexual function, and that using dienogest in the long term to treat endometriosis-related pelvic pain is able to improve QOL and sexual function. Fritzer and Hudelist (2017) reported that dyspareunia, quality of sex life, and relationships between sexual partners were improved after surgery for endometriosis. Clinically, in the nursing process for patients with endometriosis, dyspareunia improvement should be further followed up to improve the QOL of patients.

## Conclusion

To sum up, this study has found that the QOL of patients with endometriosis is negatively correlated with anxiety and depression for the first time in China, alerting medical staff to pay more attention to the correlation of QOL with unhealthy emotions among patients with endometriosis. At the same time, it has made in-depth exploration and analysis on relevant influencing factors, so as to seek relevant solutions and provide specific nursing measures for relevant patients. The study encourages active involvement of family members and reckons that it is very important to promote relevant support from family members, enhance patients’ confidence, and improve patients’ prognosis, which may provide a reference for relevant clinical nursing.

There are also some limitations in this study: 1. The questionnaire by Self-Rating Depression Scale (SDS) adopted in this study is a self-rating scale; thus, the results may be greatly influenced by the subjectivity of the subjects. In the future, other rating scales may be used for further evaluation, thus increasing the reliability of relevant appraisal. 2. This study merely included patients with endometriosis treated at the First Affiliated Hospital of Sun Yat-sen University; therefore, relevant conclusions need to be further verified by multi-center studies.

## Data Availability Statement

The original contributions presented in the study are included in the article/Supplementary Material, further inquiries can be directed to the corresponding authors.

## Ethics Statement

The studies involving human participants were reviewed and approved by the Ethics Committee of the First Affiliated Hospital of Sun Yat-sen University. The patients/participants provided their written informed consent to participate in this study. Written informed consent was obtained from the individual(s) for the publication of any potentially identifiable images or data included in this article.

## Author Contributions

GH and JC: conceptualization, methodology, investigation, formal analysis, and writing—original draft. ZP: data curation and writing—original draft. KF: data curation and investigation. CL and XZ: conceptualization, resources, supervision, and writing—review and editing. All authors contributed to the article and approved the submitted version.

## Conflict of Interest

The authors declare that the research was conducted in the absence of any commercial or financial relationships that could be construed as a potential conflict of interest.

## Publisher’s Note

All claims expressed in this article are solely those of the authors and do not necessarily represent those of their affiliated organizations, or those of the publisher, the editors and the reviewers. Any product that may be evaluated in this article, or claim that may be made by its manufacturer, is not guaranteed or endorsed by the publisher.
